# Supplementary material for: Emerging Prognostic and Predictive Significance of Stress Keratin 17 in HPV-Associated and Non HPV-Associated Human Cancers: A Scoping Review
Source: Viruses. 2023 Nov 25;15(12):2320. doi: 10.3390/v15122320 (PMC10748233; doi:10.3390/v15122320)
Supplement: Supplementary file 1 [file viruses-15-02320-s001.zip › Supplemental Data S1.pdf]

PubMed
845 Results

(“Keratin-17”[mesh] OR "keratin 17"[tiab:~7] OR "keratins 17"[tiab:~7] OR "cytokeratin 17"[tiab:~7] OR "cytokeratins 17"[tiab:~7] OR “CK 17”[tiab] OR CK17[tiab] OR “K 17”[tiab] OR K17[tiab] OR “KRT 17”[tiab] OR KRT17[tiab]) AND

("Neoplasms"[Mesh] OR neoplas*[tiab] OR cancer*[tiab] OR malignan*[tiab] OR metasta*[tiab] OR oncolog*[tiab] OR myeloma*[tiab] OR sarcoma*[tiab] OR osteosarcoma*[tiab] OR adenocarcino*[tiab] OR carcino*[tiab] OR adenom*[tiab] OR leukemia*[tiab] OR leukaemia*[tiab] OR lymphoma*[tiab] OR melanoma*[tiab] OR tumor*[tiab] OR tumour*[tiab]) NOT ("Animals"[mesh] NOT ("Animals"[mesh] AND "Humans"[mesh]))

Embase via Scopus (Elsevier)
1,196 Results

((TITLE-ABS-KEY(“CK 17” OR CK17 OR “K 17” OR K17 OR “KRT 17” OR KRT17 OR ((keratin* OR cytokeratin*) W/7 (17)))) AND (TITLE-ABS-KEY(neoplas* OR cancer* OR malignan* OR metasta* OR oncolog* OR myeloma* OR sarcoma* OR osteosarcoma* OR adenocarcino* OR carcino* OR adenom* OR leukemia* OR leukaemia* OR lymphoma* OR melanoma* OR tumor* OR tumour*)) AND NOT (INDEXTERMS((animal* OR nonhuman) AND NOT ((animal* OR nonhuman*) AND (humans))))) AND INDEX(embase)

Web of Science Core Collection (Clarivate)
1020 Results
(Core Collection Indexes: Science Citation Index Expanded, Social Sciences Citation Index, Arts & Humanities Citation Index, Conference Proceedings Citation Index – Science, Conference Proceedings Citation Index – Social Science & Humanities, Book Citation Index – Science, Book Citation Index – Social Sciences & Humanities, Emerging Sources Citation Index, Current Chemical Reactions, Index Chemicus)

(TS=(“CK 17” OR CK17 OR “K 17” OR K17 OR “KRT 17” OR KRT17 OR ((keratin* OR cytokeratin*) NEAR/7 (17)))) AND (TS=(neoplas* OR cancer* OR malignan* OR metasta* OR oncolog* OR myeloma* OR sarcoma* OR osteosarcoma* OR adenocarcino* OR carcino* OR adenom* OR leukemia* OR leukaemia* OR lymphoma* OR melanoma* OR tumor* OR tumour*))

Web of Science (Clarivate)
6 Results
Preprint Citation Index

(TS=(“CK 17” OR CK17 OR “K 17” OR K17 OR “KRT 17” OR KRT17 OR ((keratin* OR cytokeratin*) NEAR/7 (17)))) AND (TS=(neoplas* OR cancer* OR malignan* OR metasta* OR oncolog* OR myeloma* OR

sarcoma* OR osteosarcoma* OR adenocarcino* OR carcino* OR adenom* OR leukemia* OR leukaemia* OR lymphoma* OR melanoma* OR tumor* OR tumour*))

Cochrane CENTRAL via Cochrane Library (Wiley)
11 Results

("CK 17" OR CK17 OR "K 17" OR K17 OR "KRT 17" OR KRT17 OR ((keratin* OR cytokeratin*) NEAR/7 (17))):ti,ab,kw AND (neoplas* OR cancer* OR malignan* OR metasta* OR oncolog* OR myeloma* OR sarcoma* OR osteosarcoma* OR adenocarcino* OR carcino* OR adenom* OR leukemia* OR leukaemia* OR lymphoma* OR melanoma* OR tumor* OR tumour*):ti,ab,kw

---

Google Scholar
First 200 results, sorted by relevancy
"keratin 17"|"cytokeratin 17"|"ck 17"|ck17|"k 17"|k17|"KRT 17"|KRT17
adenocarcinoma|adenocarcinomas|carcinoma|neoplasm|neoplasms|neoplastic|cancer|cancers|cancerous|malignant|malignancy|malignancies|metastatic|metastasis|tumor|tumors|tumour|tumours

---
